# Types of social participation and psychological distress in Japanese older adults: A five-year cohort study

**DOI:** 10.1371/journal.pone.0175392

**Published:** 2017-04-07

**Authors:** Shiho Amagasa, Noritoshi Fukushima, Hiroyuki Kikuchi, Koichiro Oka, Tomoko Takamiya, Yuko Odagiri, Shigeru Inoue

**Affiliations:** 1 Department of Preventive Medicine and Public Health, Tokyo Medical University, Shinjuku, Tokyo, Japan; 2 Faculty of Sports Sciences, Waseda University, Tokorozawa, Saitama, Japan; Hamamatsu Ika Daigaku, JAPAN

## Abstract

**Introduction:**

The most effective type of social participation against psychological distress in older adults is not well documented. The aim of this study was to examine whether different types of social participation are associated with changes in psychological distress level in older men and women in Japan.

**Methods:**

Two thousand seven hundred community-dwelling older adults (aged 65–74 years, 50% women) were randomly selected from the resident registry of three cities. Of these, participants who reported social participation and psychological distress level in the baseline survey in 2010 were followed up. Psychological distress was evaluated based on K6 scales at baseline and follow-up (in 2015). Social participation level was examined using question items from the National Health and Nutrition Survey in Japan. Exploratory factor analysis was used to derive the underlying factor structure. Multiple linear regression analysis was used to examine the association between social participation and changes in psychological distress level after adjusting for covariates stratified by both gender and age group or living arrangement.

**Results:**

Data from 825 community-dwelling older adults (45.3% women) were analyzed. Social participation was categorized into two types using factor analysis: community involvement (volunteer activities, community events, clubs for the elderly) and individual relationship (friendship, communication with family and friends, hobbies). During the 5-year follow-up, 29.5% of participants reported a deterioration in psychological distress. Higher community involvement was independently associated with lower risk of psychological distress for older women (*β* = 0.099, p = 0.047), whereas there were no associations with individual relationship for either gender. Furthermore, in older women living with others, higher community involvement was also associated with lower risk of psychological distress (*β* = 0.110, p = 0.048).

**Conclusion:**

Community involvement provides older women with mental health benefits regardless of individual relationship level. Promoting community involvement may be an effective strategy for healthy mental aging.

## Introduction

Psychological distress is generally defined as a state of emotional suffering characterized by depressive symptoms and anxiety [1, 2]. Several studies have shown associations between psychological distress and increased risk of mortality [3–6], cardiovascular disease [7, 8], and hypertension [9]. The Global Burden of Disease (GBD) Study 2010 ranked major mental disorders as a leading cause of burden [10, 11]. In Japan, the number of older adult patients (≥65 years old) with mood or anxiety disorders has been increasing with the growing proportion of older adults, reaching 340,000 in 2014 (men: 102,000, women: 238,000) [12]. Despite both individual and social burdens of psychological distress, there is still limited information identifying its potential causes and interventions that may help in preventing psychological distress in older adults.

Social participation is a key component of successful and healthy aging in response to concerns about population aging. Previous studies have shown that older adults’ social participation is associated beneficially with mental health [13, 14], as well as quality of life (QOL) [15], cognitive functioning [16], lower mortality [17] and morbidity [18]. Several studies have found that active engagement in volunteering, religious activities, and clubs are associated with better mental health and reduced levels of depressive symptoms [14, 19–21]. Furthermore, it is reported that the impact of social participation on mental health differs by gender [22–24]; one study, for example, indicates that frequent participation at church is related to low prevalence of depressive symptoms in older women, while the opposite relationship was found in older men [22]. Another study in a Japanese population suggested that lack of participation in community sports or exercise activities was negatively associated with psychological distress in older women, but not associated in older men [24]. Since limited types of social participation have been focused on, comprehensive research related to social participation is needed.

Many similar concepts such as social engagement, social involvement, social capital, social network, social integration, and social gathering have been used interchangeably with social participation [25]. In addition to there being no agreed upon definition, social participation has included a variety of activities such as individual-based (e.g. hobby, neighborhood relationship) or community-based (e.g. local event, volunteer, senior center, religious) activities [25]. Therefore, the most effective type of social participation against poor mental health, especially for psychological distress in older adults, is not well documented. We examined whether different types of social participation were associated with changes in psychological distress level in community-dwelling Japanese older men and women.

## Methods

### Participants and data collection

This population-based, cohort study was conducted using a postal survey in 2010 (baseline) and 2015 (follow-up). We included three Japanese cities of various population densities; Bunkyo city, Fuchu city and Oyama city. Bunkyo is an urban city in the Tokyo metropolitan area, Fuchu is a suburban city of Tokyo within commuting distance to the central business district, and Oyama in Shizuoka prefecture is a typical small rural city located about 80 km west of Tokyo. The study sample aged 65 to 74 years (N = 2,700) was randomly selected from the resident register of each municipality, which contains all individual information of residents in each city. The details of the sampling process for the 2010 survey and the locations, areas, population sizes, and population densities of each area are described in a previous study [26].

Ethical approval for the study was obtained from the Tokyo Medical University Ethics Committee prior to the survey in 2010 (No. 1273) and in 2015 (No. 2898). All participants signed the consent form before answering the questionnaire.

**Fig 1:**
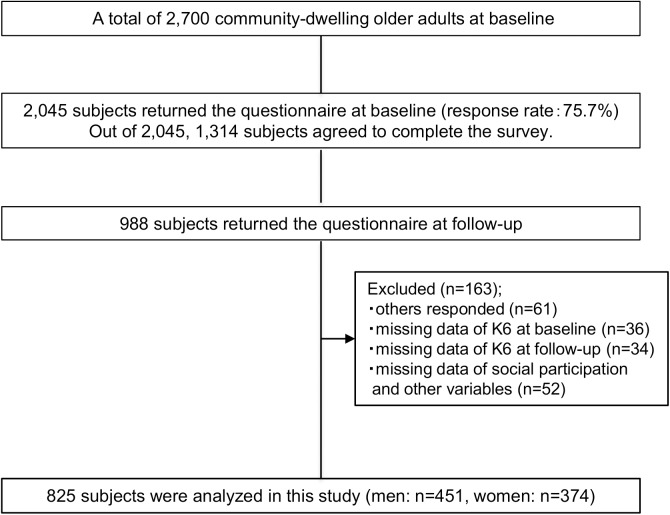
Flow diagram of study participant enrollment.

Fig 1 is a flow diagram of participant enrollment. Of 2,700 older adults invited to participate, 2,045 people returned the questionnaire at baseline (response rate 75.7%) and 1,314 of them agreed to complete the survey at follow-up. We provided 1,314 potential participants advance notification of the intention to send a questionnaire one month before the survey at follow-up. In this process, we excluded 104 people because of refusal (n = 6), address unknown (n = 93) and death (n = 5). Therefore, we sent a questionnaire to 1,210 participants at follow-up. Though 988 of them returned the questionnaire at follow-up, we included 927 respondents who had answered the questionnaire by themselves. Then, we excluded 102 due to missing data of psychological distress level (n = 36 at baseline and n = 34 at follow-up) and missing data of social participation and other variables (n = 52). Therefore, there were 825 (451 men, 374 women) final participants.

### Measures

#### Dependent variable

Psychological distress was measured using the Kessler 6 (K6) scale [27] at both baseline and follow-up. The scale assesses how often respondents had experienced the following depressive symptoms over the last 30 days; feeling nervous, hopeless, restless or fidgety, so depressed that nothing could cheer you up, everything was an effort, and worthless [27]. Each of the six items are scaled from 0 (none of the time) to 4 (all of the time), and the total score (0–24) is used as an index of psychological distress level (i.e. higher scores represented more severe psychological distress). Good validity and reliability for screening serious mental illness in the general population [27, 28] have been demonstrated and the K6 has been translated into Japanese [29].

In the present study, deterioration in psychological distress was defined as any change from the baseline score to the score at follow-up. This was calculated by subtracting the baseline K6 score (0–24) from the follow-up K6 score (0–24), where the calculated value indicated -24 to -1: improvement, 0: unchanged, 1 to 24: deterioration, respectively (i.e. a lower value represents improvement of psychological distress).

#### Independent variables

Social participation level was examined at baseline using question items from the National Health and Nutrition Survey in Japan in 2006. Social participation included six types of activities: hobby, friendship, clubs for the elderly, volunteer activities, community events and communication with family members and friends. Each social participation level ranged from 1 to 3 points (1: frequently, 2: sometimes, 3: seldom).

#### Covariates

Information about age, gender, and residential area were obtained from the resident registry of each municipality. The following factors acquired through using self-reported questionnaires were relevant confounders for statistical control: living arrangement, working status, Body Mass Index (BMI), physical health, smoking, and drinking. Physical functioning was measured with one item from the Short Form-8 (SF-8) [30], which is commonly used to assess physical health: “during the past 4 weeks, how much did physical health problems limit your usual physical activities (such as transfers or going places)?” The answers were categorized into “good” (not at all, very little, and somewhat) and “bad” (quite a lot and could not do physical activities). Smoking and drinking status was examined using question items from the National Health and Nutrition Survey in Japan.

### Statistical analyses

A chi-squared test was conducted to determine whether there was a significant association between categorical variables. For the continuous variables, data comparisons between genders were tested with Mann-Whitney U tests. To identify types of social participation, we then conducted exploratory factor analysis with principal axis extraction and varimax Kaiser normalization. Cronbach’s alpha was calculated to examine the scale's internal consistency. We used eigenvalues greater than one in the factor analysis to retain factors. Multiple regression analysis was used to examine the association between social participation level and change in psychological distress, calculating the adjusted *β* (standardized coefficients) and 95% confidence intervals (CIs) of *β*, stratified by gender. Additionally, multiple regression analyses stratified by age and living arrangement were performed. Each categorical covariate was classified as follows; residential area: “Bunkyo”, “Fuchu”, or “Oyama”; living arrangement: “with others” or “alone”, working status: “working with income” or “not working”, physical health: “good” or “bad”, smoking: “yes” or “no” and drinking: “yes” or “no”. We also conducted multiple regression analyses where only older adults without psychological distress (<5 points) at baseline were included based on a recommended cutoff point for screening mood/anxiety disorder in the general population [31, 32].

Three models were performed by gender, simultaneously controlling for potential confounders; model 1: age, area, living arrangement, and working, model 2: BMI, physical functioning, smoking, and drinking were added to model 1, and model 3: each type of social participation generated after the factor analysis was mutually adjusted (i.e. entering all variables). All covariates were entered into the model at the same time since there was no multicollinearity (< 5 Variance Inflation Factor). The significance level was set at p < .05. All statistical analyses were conducted using IBM SPSS Statistics version 21 (SPSS Inc., Tokyo, Japan).

## Results

### Participant characteristics

The mean age at baseline was 69.3±2.9 years in men and 69.3±2.9 years in women. Fewer than half of the respondents were women (45.3%), and most of the study population was living with others (men: 92.2%, women: 86.9%) and had good physical functioning (men: 97.1%, women: 93.6%) (Table 1). The median (25%, 75%) K6 scores at baseline were 1.0 (0.0, 3.0) in men and 1.0 (0.0, 4.0) in women. There were statistically significant baseline group differences in living arrangement, working, smoking, drinking, physical functioning, BMI, and individual relationship level (p<0.05). During the 5-year follow-up, 29.5% (men: 27.7%, women: 31.6%) of participants reported worsening of psychological distress, while 33.9% (men: 33.3%, women: 34.8%) improved and 36.6% (men: 39.0%, women: 33.7%) did not change (results are not shown in the table). There was no significant gender difference in pattern of change in K6 score (chi-squared test, p = 0.151).

**Table 1:** Descriptive data of participants at baseline (2010).

	Men (n = 451)		Women (n = 374)		
	n (%) / median (25%, 75%)		n (%) / median (25%, 75%)		p-value
**Age**					0.716^a^
65–69 years	239	(53.0)	201	(53.7)	
70–74 years	212	(47.0)	173	(46.3)	
**Area**					0.104 ^a^
Bunkyo	144	(31.9)	130	(34.8)	
Fuchu	159	(35.3)	106	(28.3)	
Oyama	148	(32.8)	138	(36.9)	
**Living arrangement**					0.015 ^a^
with others	416	(92.2)	325	(86.9)	
alone	35	(7.8)	49	(13.1)	
**Working with income**					<0.001 ^a^
yes	245	(54.3)	123	(32.9)	
no	206	(45.7)	251	(67.1)	
**Smoking**					<0.001 ^a^
yes	104	(23.1)	18	(4.8)	
no	347	(76.9)	356	(95.2)	
**Drinking**					<0.001 ^a^
yes	319	(70.7)	104	(27.8)	
no	132	(29.3)	270	(72.2)	
**Physical functioning**					0.018 ^a^
good	438	(97.1)	350	(93.6)	
bad	13	(2.9)	24	(6.4)	
**Body Mass Index (BMI)**					<0.005 ^a^
< 25 (kg/m^2^)	345	(76.5)	316	(84.5)	
≥ 25 (kg/m^2^)	106	(23.5)	58	(15.5)	
**Psychological distress (K6) score**					0.058 ^a^
< 5 (point)	379	(84.0)	295	(78.9)	
≥ 5 (point)	72	(16.0)	79	(21.1)	
**Social participation level (3–9 points)**					
individual relationship (lower score: frequently)	5.5	(4.4, 6.6)	4.9	(3.8, 6.0)	<0.001^b^
community involvement (lower score: frequently)	7.8	(6.3, 8.7)	7.6	(6.3, 8.6)	0.223 ^b^

^a^P-value was calculated using chi-squared tests.

^b^P-value was calculated using Mann-Whitney U tests.

### Result of the factor analysis

As a result of the factor analysis, two social participation types were derived with eigenvalues greater than one: community involvement (volunteer activities, community events, clubs for the elderly) (Cronbach’s α = 0.63), and individual relationship (hobby, friendship, communication with family members and friends) (Cronbach’s α = 0.54). The rotated factor loadings are presented in Table 2.

**Table 2:** Rotated factor loadings (varimax rotation).

	Men		Women	
	Factor 1	Factor 2	Factor 1	Factor 2
Variables	community involvement	individual relationship	community involvement	individual relationship
Clubs for the elderly	0.698	-0.016	0.738	-0.083
Volunteer activities	0.801	0.201	0.606	0.354
Community events	0.742	0.125	0.791	0.080
Hobby	0.131	0.610	0.027	0.746
Friendship	0.101	0.747	0.196	0.569
Communication with family and friends	0.022	0.717	-0.022	0.647

### Results of the multiple regression analysis

In the multiple regression analysis, there were no statistically significant associations between social participation and risk of psychological distress in model 1 and 2 for both genders (Table 3). However, in model 3 (i.e. fully adjusted model), higher community involvement level was independently associated with lower risk of psychological distress for older women (*β* = 0.099, β95%CI = 0.003–0.443, p = 0.047), whereas individual relationship level was not related to either gender. These results did not change when participants who had K6 scores ≥5 points at baseline were excluded from the analyses. There was no significant interaction between men and women, or younger and elderly. The impacts of covariates on psychological distress in older adults by age group are shown in S1 Table.

**Table 3:** Association of social participation level with psychological distress in community-dwelling older adults by age group.

		Model 1^a^				Model 2 ^b^				Model 3 ^c^			
		*β*	*β* 95%CI		p-value	*β*	*β* 95%CI		p-value	*β*	*β* 95%CI		p-value
**Men**	**Community involvement**												
	total	-0.021	-0.217	0.141	0.680	0.013	-0.144	0.189	0.790	0.033	-0.119	0.237	0.518
	65–69 years	-0.012	-0.254	0.213	0.861	0.014	-0.198	0.247	0.826	0.020	-0.201	0.270	0.775
	70–74 years	-0.014	-0.304	0.253	0.856	0.007	-0.242	0.267	0.923	0.043	-0.198	0.360	0.567
	**Individual relationship**												
	total	-0.089	-0.360	0.008	0.061	-0.050	-0.276	0.077	0.267	-0.053	-0.291	0.084	0.277
	65–69 years	-0.062	-0.341	0.117	0.338	-0.035	-0.288	0.160	0.574	-0.019	-0.265	0.198	0.775
	70–74 years	-0.121	-0.562	0.038	0.087	-0.060	-0.417	0.156	0.370	-0.083	-0.492	0.134	0.261
**Women**	**Community involvement**												
	total	0.050	-0.128	0.352	0.359	0.082	-0.025	0.392	0.084	0.099	0.003	0.443	0.047
	65–69 years	0.057	-0.220	0.511	0.434	0.045	-0.197	0.423	0.472	0.048	-0.216	0.457	0.481
	70–74 years	0.032	-0.274	0.408	0.699	0.087	-0.126	0.488	0.247	0.112	-0.088	0.555	0.154
	**Individual relationship**												
	total	-0.067	-0.404	0.085	0.199	-0.013	-0.237	0.178	0.428	-0.029	-0.292	0.155	0.548
	65–69 years	-0.079	-0.491	0.133	0.259	-0.003	-0.268	0.255	0.559	-0.007	-0.307	0.273	0.809
	70–74 years	-0.051	-0.529	0.269	0.521	-0.014	-0.385	0.313	0.738	-0.023	-0.431	0.310	0.749

^a^Model 1: Adjusted for age, area (Bunkyo/ Fuchu/ Oyama), living arrangement (with others/ alone), working (yes/ no)

^b^Model 2: Adjusted for age, area (Bunkyo/ Fuchu/ Oyama), living arrangement (with others/ alone), working (yes/ no), BMI, physical functioning (good/ bad), smoking (yes/ no), drinking (yes/ no)

^c^Model 3: Adjusted for age, area (Bunkyo/ Fuchu/ Oyama), living arrangement (with others/ alone), working (yes/ no), BMI, physical functioning (good/ bad), smoking (yes/ no), drinking (yes/ no), community involvement or individual relationship

Table 4 represents the adjusted β for change of psychological distress level by living arrangement. In older women living alone, higher individual relationship was associated with lower risk of psychological distress (β = 0.258, β 95%CI = 0.052–1.396, p = 0.035) and this association remained significant after adjustment for community involvement (β = 0.249, β 95%CI = 0.033–1.363, p = 0.040). On the other hand, in older women living with others, higher community involvement was independently associated with lower risk of psychological distress (β = 0.110, β 95%CI = 0.002–0.468, p = 0.048). S2 Table provides the impacts of covariates on psychological distress in older adults by living arrangement.

**Table 4:** Association of social participation level with psychological distress in community-dwelling older adults by living arrangement.

		Model 1^a^				Model 2 ^b^				Model 3 ^c^			
		*β*	*β* 95%CI		p-value	*β*	*β* 95%CI		p-value	*β*	*β* 95%CI		p-value
**Men**	**Community involvement**												
	living with others	-0.014	-0.208	0.157	0.784	0.007	-0.160	0.185	0.886	0.024	-0.140	0.228	0.640
	living alone	0.032	-0.545	0.655	0.853	0.055	-0.433	0.622	0.716	0.016	-0.598	0.653	0.928
	**Individual relationship**												
	living with others	-0.114	-0.420	-0.040	0.018	-0.048	-0.283	0.088	0.300	-0.046	-0.288	0.103	0.352
	living alone	0.068	-0.471	0.694	0.699	0.086	-0.373	0.656	0.577	0.078	-0.484	0.739	0.671
**Women**	**Community involvement**												
	living with others	0.017	-0.232	0.294	0.597	0.065	-0.080	0.357	0.213	0.110	0.002	0.468	0.048
	living alone	0.139	-0.545	1.308	0.411	0.200	-0.199	1.300	0.146	0.185	-0.212	1.229	0.162
	**Individual relationship**												
	living with others	-0.165	-0.613	-0.134	0.002	-0.078	-0.394	0.043	0.116	-0.102	-0.473	0.009	0.059
	living alone	0.169	-0.372	1.323	0.265	0.258	0.052	1.396	0.035	0.249	0.033	1.363	0.040

^a^Model 1: Adjusted for age, area (Bunkyo/Fuchu/Oyama), working (yes/no)

^b^Model 2: Adjusted for age, area (Bunkyo/Fuchu/Oyama), working (yes/no), BMI, physical functioning (good/bad), smoking (yes/no), drinking (yes/no)

^c^Model 3: Adjusted for age, area (Bunkyo/Fuchu/Oyama), working (yes/no), BMI, physical functioning (good/bad), smoking (yes/no), drinking (yes/no), community involvement or individual relationship

## Discussion

Our study explores the associations between different types of social participation and changes in psychological distress level in community-dwelling Japanese older adults. Higher community involvement was independently associated with lower risk of psychological distress in community-dwelling Japanese older women. However, both community involvement and individual relationship were not related to risk of psychological distress in older men.

In terms of gender differences, previous studies have indicated a gender difference in the relationship between social participation and mental health [22, 23, 33–35]. Our result was consistent with some studies suggesting that women gain more benefit from their social participation [22, 23, 34, 35]. In the current study, we found community involvement was associated beneficially with psychological distress for older women. Our findings correspond to Kuriyama et al.’s report that no participation in community activities was negatively associated with mental health in older women (≥65 years) [24]. This is probably because community involvement provides a sense of meaning in people’s lives as well as fulfillment and opportunity for social support [36–39]. A previous study showed, for example, that volunteering improved mental health by enhancing the participants’ range of social networks [38]. Atkins et al. reported that lower levels of social support were related to higher psychological distress [40]. Moreover, women, more than men, tend to build relationships from their wide and diverse networks, and women may be inclined to receive positive benefits from community involvement. Norton et al. reported that frequent religious engagement was related to a reduced prevalence of depression; our findings partly support Norton et al.’s argument [22] because religious involvement is considered to be one type of community involvement.

In the present study, individual relationship did not have a favorable association on psychological distress in overall older men and women although previous studies have reported the link between fewer close relationship and depressive symptoms [36, 41]. Older adults who have higher level of personal relationships with others (e.g. family, relatives, friends, and neighbors) may be more likely to face problems and traumatic/stressful events such as death of a loved one and someone’s death from disease, and thus these predispose them to feel emotionally close and depressive [36]. As our finding shows, individual relationship which is fundamental of social participation [25] may be more important for older women living alone, who are likely to have less income and to be in poor health compared to those living with others [42]. A previous study by Victor et al. [43] showed that older women living alone were more likely than those with others to experience feeling of social isolation partly because of a lack of social network including emotional support. Improved social relationships were connected with reduced level of loneliness [43] which was associated with depression [44, 45]. Compared to community involvement, individual relationship is more informal and therefore older women living alone can be dependent principally on individual relationship for emotional support. Individual relationship may have had a protective role against psychological distress in older women living alone.

### Strengths and limitations

Our study has several strengths. First, to our knowledge, this is the first cohort study to examine the association between social participation and change of psychological distress level in community-dwelling older adults. Our study permits a causal interpretation of our findings based on its follow-up design. Second, we performed the random sampling of participants from each included city at baseline. Third, older adults from cities with different population densities were included in this study.

Some limitations of our study should be considered. First, some respondents were excluded from our analysis due to missing data. Generally, missing responses and data may tend to be higher in older adults with more severe psychological stress and lower social participation level, which may have biased the findings. Second, the generalization of the results of our study to other populations may be somewhat difficult. Third, we cannot deny the possibility of residual confounding factors as there could have been some unmeasured variables that influenced the association between social participation and changes in psychological distress level. For example, our study did not examine whether participants had a social role in social organizations. Takagi et al. reported that older men who occupied leadership positions in organizations rated better mental health [34]; therefore, additional adjustment for social role may change this association. In addition, it is possible that certain personality traits are connected with both social participation and the occurrence of mental health [41]. Finally, social participation and psychological distress level were evaluated using self-reported information and thus the measurements themselves may have reporting bias. In present analyses, we included participants who had different physiological distress level, and thus individuals with high K6 score compared to those with low one might have tended to make more negative interpretation of social participation level [46].

## Conclusion

Change in psychological distress level was found to be associated with certain types of social participation in older adults. Community involvement provides older women with mental health benefits regardless of individual relationship level. Promoting community involvement may be an effective strategy for healthy mental aging.

## Supporting information

S1 Table Impacts of covariates on psychological distress in older adults by age group. (DOCX)

S2 Table Impacts of covariates on psychological distress in older adults by living arrangement. (DOCX)
