# Supplementary material for: Types of social participation and psychological distress in Japanese older adults: A five-year cohort study
Source: PLoS One. 2017 Apr 7;12(4):e0175392. doi: 10.1371/journal.pone.0175392 (PMC5384679; doi:10.1371/journal.pone.0175392)
Supplement: S1 Table — (DOCX) [file pone.0175392.s001.docx]

S1 Table. Impacts of covariates on psychological distress in older adults by age group

|  |  | Total | | | |  | 65-69 years | | | |  | 70-74 years | | | | |  |
| --- | --- | --- | --- | --- | --- | --- | --- | --- | --- | --- | --- | --- | --- | --- | --- | --- | --- |
|  |  | β | β 95%CI | | p-value |  | β | β 95%CI | | p-value |  | β | β 95%CI | | p-value | |  |
| **Men** | **Age** | 0.037 | -0.053 | 0.123 | 0.438 |  | 0.168 | 0.078 | 0.523 | 0.008 |  | -0.030 | -0.330 | 0.214 | 0.675 | |  |
|  | **Area** |  |  |  |  |  |  |  |  |  |  |  |  |  |  | |  |
|  | Oyama | ref. | | | |  | ref. | | | |  | ref. | | | | |  |
|  | Bunkyo | -0.008 | -0.710 | 0.619 | 0.894 |  | 0.067 | -0.445 | 1.163 | 0.379 |  | -0.069 | -1.573 | 0.685 | 0.439 | |  |
|  | Fuchu | -0.015 | -0.710 | 0.536 | 0.784 |  | 0.017 | -0.690 | 0.866 | 0.824 |  | -0.048 | -1.321 | 0.744 | 0.583 | |  |
|  | **Living arrangement** |  |  |  |  |  |  |  |  |  |  |  |  |  |  | |  |
|  | yes | ref. | | | |  | ref. | | | |  | ref. | | | | |  |
|  | no | 0.003 | -0.888 | 0.950 | 0.947 |  | -0.057 | -1.916 | 0.730 | 0.378 |  | 0.058 | -0.818 | 1.956 | 0.419 | |  |
|  | **Working** |  |  |  |  |  |  |  |  |  |  |  |  |  |  |  |  |
|  | yes | ref. | | | |  | ref. | | | |  | ref. | | | | | |
|  | no | 0.007 | -0.470 | 0.541 | 0.890 |  | 0.061 | -0.336 | 0.961 | 0.343 |  | -0.029 | -0.993 | 0.648 | 0.679 | |  |
|  | **BMI** | -0.012 | -0.100 | 0.076 | 0.789 |  | 0.026 | -0.088 | 0.134 | 0.683 |  | -0.075 | -0.226 | 0.069 | 0.294 | |  |
|  | **Drinking** |  |  |  |  |  |  |  |  |  |  |  |  |  |  | |  |
|  | yes | ref. | | | |  | ref. | | | |  | ref. | | | | |  |
|  | no | -0.049 | -0.835 | 0.250 | 0.290 |  | -0.059 | -1.016 | 0.367 | 0.356 |  | -0.067 | -1.331 | 0.472 | 0.348 | |  |
|  | **Smoking** |  |  |  |  |  |  |  |  |  |  |  |  |  |  | |  |
|  | yes | ref. | | | |  | ref. | | | |  | ref. | | | | |  |
|  | no | -0.016 | -0.695 | 0.494 | 0.741 |  | -0.052 | -1.004 | 0.422 | 0.422 |  | 0.018 | -0.917 | 1.182 | 0.804 | |  |
|  | **Physical functioning** |  |  |  |  |  |  |  |  |  |  |  |  |  |  | |  |
|  | good | ref. | | | |  | ref. | | | |  | ref. | | | | |  |
|  | bad | 0.067 | -0.438 | 2.859 | 0.150 |  | 0.095 | -0.434 | 3.164 | 0.136 |  | 0.030 | -2.651 | 4.118 | 0.670 | |  |
|  |  |  |  |  |  |  |  |  |  |  |  |  |  |  |  | |  |
| **Women** | **Age** | 0.065 | -0.032 | 0.175 | 0.175 |  | 0.049 | -0.175 | 0.405 | 0.434 |  | -0.002 | -0.300 | 0.292 | 0.978 | |  |
|  | **Area** |  |  |  |  |  |  |  |  |  |  |  |  |  |  | |  |
|  | Oyama | ref. | | | |  | ref. | | | |  | ref. | | | | | |
|  | Bunkyo | -0.092 | -1.343 | 0.108 | 0.095 |  | -0.119 | -1.801 | 0.169 | 0.104 |  | -0.047 | -1.436 | 0.825 | 0.594 | |  |
|  | Fuchu | -0.073 | -1.258 | 0.224 | 0.171 |  | -0.075 | -1.652 | 0.484 | 0.282 |  | -0.042 | -1.365 | 0.823 | 0.626 | |  |
|  | **Living arrangement** |  |  |  |  |  |  |  |  |  |  |  |  |  |  | |  |
|  | yes | ref. | | | |  | ref. | | | |  | ref. | | | | | |
|  | no | -0.092 | -1.723 | -0.011 | 0.047 |  | -0.133 | -2.777 | -0.093 | 0.036 |  | -0.051 | -1.584 | 0.755 | 0.485 | |  |
|  | **Working** |  |  |  |  |  |  |  |  |  |  |  |  |  |  | |  |
|  | yes | ref. | | | |  | ref. | | | |  | ref. | | | | | |
|  | no | -0.051 | -0.965 | 0.269 | 0.269 |  | -0.081 | -1.364 | 0.279 | 0.194 |  | -0.019 | -1.190 | 0.909 | 0.791 | |  |
|  | **BMI** | -0.073 | -0.137 | 0.014 | 0.110 |  | -0.136 | -0.290 | -0.015 | 0.030 |  | -0.049 | -0.127 | 0.061 | 0.489 | |  |
|  | **Drinking** |  |  |  |  |  |  |  |  |  |  |  |  |  |  | |  |
|  | yes | ref. | | | |  | ref. | | | |  | ref. | | | | |  |
|  | no | -0.133 | -1.625 | -0.278 | 0.006 |  | -0.166 | -2.079 | -0.264 | 0.012 |  | -0.080 | -1.661 | 0.485 | 0.280 | |  |
|  | **Smoking** |  |  |  |  |  |  |  |  |  |  |  |  |  |  | |  |
|  | yes | ref. | | | |  | ref. | | | |  | ref. | | | | |  |
|  | no | -0.030 | -1.813 | 0.917 | 0.519 |  | -0.025 | -2.063 | 1.359 | 0.685 |  | -0.033 | -3.150 | 1.984 | 0.654 | |  |
|  | **Physical functioning** |  |  |  |  |  |  |  |  |  |  |  |  |  |  | |  |
|  | good | ref. | | | |  | ref. | | | |  | ref. | | | | |  |
|  | bad | -0.103 | -2.615 | -0.167 | 0.026 |  | -0.096 | -3.308 | 0.413 | 0.127 |  | -0.118 | -3.100 | 0.316 | 0.109 | |  |

Impacts of community involvement and individual relationship on psychological distress are shown in Table 3 in the main text.
