# Supplementary material for: Types of social participation and psychological distress in Japanese older adults: A five-year cohort study
Source: PLoS One. 2017 Apr 7;12(4):e0175392. doi: 10.1371/journal.pone.0175392 (PMC5384679; doi:10.1371/journal.pone.0175392)
Supplement: S2 Table — (DOCX) [file pone.0175392.s002.docx]

S2 Table. Impacts of covariates on psychological distress in older adults by living arrangement

|  |  | Living with others | | | |  | Living alone | | | |
| --- | --- | --- | --- | --- | --- | --- | --- | --- | --- | --- |
|  |  | β | β 95%CI | | p-value |  | β | β 95%CI | | p-value |
| **Men** | **Age** | 0.036 | -0.058 | 0.127 | 0.463 |  | 0.051 | -0.278 | 0.349 | 0.819 |
|  | **Area** |  |  |  |  |  |  |  |  |  |
|  | Oyama | ref. | | | |  | ref. | | | |
|  | Bunkyo | -0.002 | -0.704 | 0.678 | 0.971 |  | 0.027 | -2.174 | 2.414 | 0.915 |
|  | Fuchu | -0.015 | -0.733 | 0.565 | 0.799 |  | -0.087 | -2.303 | 1.565 | 0.697 |
|  | **Working** |  |  |  |  |  |  |  |  |  |
|  | yes | ref. | | | |  | ref. | | | |
|  | no | 0.007 | -0.488 | 0.569 | 0.881 |  | 0.029 | -1.408 | 1.651 | 0.871 |
|  | **BMI** | 0.011 | -0.080 | 0.103 | 0.811 |  | -0.503 | -0.680 | -0.066 | 0.019 |
|  | **Drinking** |  |  |  |  |  |  |  |  |  |
|  | yes | ref. | | | |  | ref. | | | |
|  | no | -0.070 | -0.992 | 0.141 | 0.140 |  | 0.309 | -0.576 | 3.228 | 0.163 |
|  | **Smoking** |  |  |  |  |  |  |  |  |  |
|  | yes | ref. | | | |  | ref. | | | |
|  | no | -0.028 | -0.805 | 0.434 | 0.557 |  | 0.155 | -1.332 | 2.694 | 0.491 |
|  | **Physical functioning** |  |  |  |  |  |  |  |  |  |
|  | good | ref. | | | |  | ref. | | | |
|  | bad | 0.074 | -0.351 | 2.950 | 0.122 |  | 0.024 | -2.701 | 4.203 | 0.664 |
|  |  |  |  |  |  |  |  |  |  |  |
| **Women** | **Age** | 0.048 | -0.057 | 0.156 | 0.361 |  | 0.204 | -0.089 | 0.683 | 0.127 |
|  | **Area** |  |  |  |  |  |  |  |  |  |
|  | Oyama | ref. | | | |  | ref. | | | |
|  | Bunkyo | -0.106 | -1.412 | 0.065 | 0.074 |  | -0.054 | -3.434 | 2.539 | 0.763 |
|  | Fuchu | -0.082 | -1.281 | 0.202 | 0.153 |  | -0.118 | -4.911 | 2.623 | 0.542 |
|  | **Working** |  |  |  |  |  |  |  |  |  |
|  | yes | ref. | | | |  | ref. | | | |
|  | no | -0.062 | -1.028 | 0.245 | 0.227 |  | -0.078 | -3.090 | 1.699 | 0.560 |
|  | **BMI** | -0.067 | -0.169 | 0.033 | 0.186 |  | -0.096 | -0.211 | 0.099 | 0.469 |
|  | **Drinking** |  |  |  |  |  |  |  |  |  |
|  | yes | ref. | | | |  | ref. | | | |
|  | no | -0.118 | -1.504 | -0.091 | 0.027 |  | -0.179 | -4.188 | 1.091 | 0.242 |
|  | **Smoking** |  |  |  |  |  |  |  |  |  |
|  | yes | ref. | | | |  | ref. | | | |
|  | no | -0.050 | -2.104 | 0.694 | 0.322 |  | 0.051 | -5.287 | 7.453 | 0.732 |
|  | **Physical functioning** |  |  |  |  |  |  |  |  |  |
|  | good | ref. | | | |  | ref. | | | |
|  | bad | -0.037 | -1.810 | 0.842 | 0.473 |  | -0.287 | -7.718 | -0.173 | 0.041 |

Impacts of community involvement and individual relationship on psychological distress are shown in Table 4 in the main text.
